# An Efficient Content Store-Based Forwarding Scheme for Internet of Things †

**DOI:** 10.3390/s21227607

**Published:** 2021-11-16

**Authors:** Ngoc-Thanh Dinh, Younghan Kim

**Affiliations:** School of Electronic Engineering, Soongsil University, Seoul 06978, Korea; younghak@dcn.ssu.ac.kr

**Keywords:** Internet of Things, wireless sensor networks, information centric networking, forwarding scheme, content store, information objects

## Abstract

One of the main advantages of information-centric networking (ICN) is that a requested piece of content can be retrieved from a content store (CS) at any intermediate node, instead of its original content producer. In existing ICN designs, nodes forward Interest packets mainly based on forwarding information base (FIB). FIB is constructed from name prefixes registered by content producers with a list of next hops to the name prefixes. The ICN forwarding engine uses those information to forward Interest packets towards corresponding content producers. CS information of a node is currently used only for checking the availability of cached content objects at the node and is not considered in the data plane of existing ICN forwarding mechanisms. This paper highlights the importance of CS information in an ICN forwarding mechanism and enables neighbor CS information in the data plane to improve the cache hit ratio and forwarding efficiency, especially for resource-constraint Internet of Things (IoT). We propose an efficient CS-based forwarding scheme for IoT. The proposed forwarding scheme exploits CS information of neighbors to find efficient routes to forward Interest packets toward nearby nodes with corresponding cached content. For that, we carefully design an efficient way for CS information sharing using counting bloom filter. We implement the proposed scheme and compare with state-of-the-art ICN forwarding schemes in IoT. Experimental results indicate that the proposed forwarding scheme achieves a significant improvement in terms of cache hit ratio, energy efficiency, content retrieval latency, and response rate.

## 1. **Introduction**

This paper extends our preliminary version [1] to develop an efficient content store-based forwarding scheme in information centric networking. Content-centric caching and replication have shown significant benefits to improve the performance in Content Delivery Networks (CDNs) and Peer-to-Peer (P2P) [2,3] which are the first attempts to exploit availability of cheap storage and processing capabilities for efficient content retrieval. Despite the advantages of P2P and CDNs, their performance and acceptance are limited due to the operations at the application layer, the commercial administration and technologies boundaries which they apply [4]. Information-centric networking (ICN) [2,3] is proposed to address shortcomings of the traditional end-to-end communication model and of CDNs as well as P2P networking by designing a network layer protocol which focuses on information dissemination and retrieval. In ICN, routing operations are executed based on content names.

ICN is considered as a potential networking architecture for Internet of Things (IoT) [5] which consists of physical objects embedded with sensors, processing ability, communication ability and other technologies that collect and exchange data about the environment around them or the way the objects are used. The reason is that ICN had shown its efficiency and large naming space with a thin information-centric networking stack [4,6,7]. The major traffic pattern in IoT is related to information retrieval, not end-to-end communication. In addition, IoT users normally care about content itself being retrieved in a proper time manner, not where the content is stored. As a matching design, the ICN paradigm also focuses on the content itself and use content names, not the location (i.e., IP addresses) for packet forwarding.

In particular, ICN decouples the content location and the content producer from content Interest packet forwarding. Based on the location decoupling and content naming features, ICN implements in-network caching [8,9] at intermediate nodes to enhance the network performance. This is one of the main advantages of ICN because a requested content can be retrieved from the content store (CS) at any intermediate node, instead of its original content producer. In other words, content Interest packets can be satisfied faster with cached content objects to reduce the forwarding distance and improve the network performance. In this way, in-networking caching potentially helps shorten the packet latency and lower the server load, which are two critical factors in IoT because of its low-power and lossy network conditions. Therefore, in-network caching plays an important role in the ICN paradigm for IoT.

In the current design of ICN in general and named data networking (NDN), specifically [2,3], CS of a node is used to store information of cached contents. An Interest packet is forwarded based on forwarding information base (FIB) towards content producers. At each node, the Interest packet is matched with the CS of the node to check whether or not the requested content is cached at the node. If the requested content is cached at the node, the cached content is retrieved directly from the content store (CS) of the node to the requester, thus improving the network performance and reducing the network overhead. However, CS information is not utilized in the data plane of existing ICN forwarding engines [2,3] in which nodes forward Interest packets mainly based on FIB. FIB is constructed from name prefixes registered by content producers, consisting of lists containing next hops to name prefixes. This information is used to forward Interests towards corresponding content producers. CS information is currently used to check the existence of requested content at local nodes which are on the routing path toward the corresponding producer, not to route Interest packets. This may lead to inefficient Interest packet forwarding in many cases as analyzed in Section 2. For example, if some neighbor nodes that are not on the routing path toward the producer have a cache of the requested content, the forwarder would not know and still forward the request toward the producer in multiple hops. As a result, the role of content store is limited.

This paper highlights the importance of CS information in an ICN forwarding mechanism. We propose to enable neighbor CS information in the data plane for Interest packet forwarding purposes. The idea is to exploit the CS information of nodes to improve the network performance. For the purpose, we design an efficient CS-based forwarding scheme to improve the cache hit ratio and forwarding efficiency, especially for resource-constraint Internet of Things (IoT) [5] where forwarding efficiency and reducing radio communication activities are very important to save the power of IoT nodes. The proposed forwarding scheme exploits CS information of neighbors to find efficient routes to forward Interest packets toward nearby nodes with corresponding cached content. If nearby neighbors have a cached object of the requested content, the request can be satisfied faster with a cache hit which helps improve the forwarding efficiency and reduces the packet forwarding latency. For efficiency, we carefully design an efficient way for CS information sharing using counting bloom filter and neighbor CS information storing using neighbor content store tables. In particular, each node only needs to summarize a compact set of representing lists of content names cached in the CS of the node using an array of bits. We implement the proposed scheme in Contiki [6] and compare with state-of-the-art ICN forwarding schemes in IoT. Obtained experimental results show that the proposed scheme achieves a significant improvement in terms of energy efficiency, cache hit ratio, content retrieval latency, and response rate.

The rest of this paper is organized as follows: Section 2 discusses related works. Section 3 gives the overview and the detailed design of the proposed CS-based forwarding scheme. Section 4 describes our experiments and evaluation. Finally, Section 5 concludes the paper.

## 2. Related Work

Information-centric networking (ICN) names content objects and exploits content names for routing. In its core design, ICN decouples the content location and the content producer from content Interest packet forwarding. Relying on the location decoupling and content naming, ICN is designed with in-network caching [10,11] in which content objects can be stored and retrieved at intermediate nodes to enhance the network performance. We consider this feature as one of the main advantages of ICN. With this feature, content Interest packets can be satisfied with cached content objects to improve the overall network performance.

In the current design of ICN [3,6], CS of a node is used to store information of cached contents. An Interest packet is forwarded based on forwarding information base (FIB) towards corresponding content producer. At each intermediate node, the Interest packet is matched with the CS of the node to check whether or not the requested content is cached at the node. If the requested content is cached at the node, the cached content is retrieved directly from the content store (CS) of the node to the requester, thus improving the network performance and reducing the network overhead. However, CS information is not considered in in the data plane of current native ICN routing protocols (i.e., in NDN and content centric networking (CCNx)) in which Interest packets are forwarded mainly using forwarding information base (FIB). FIB is built based on name prefixes registered by content producers, lists the next hops to name prefixes, and uses that information to forward Interests towards the producers. CS information at a node is used only to check the availability of requested content at the local node on the routing path toward the producers, not to route Interest packets. In the literature as described in [4], several extended routing protocols were proposed. INFORM [12] is a dynamic forwarding mechanisms based on Q-learning while iNRR [13] is based on flooding. However, most of them use a separate content replica discovery, thus resulting in a high overhead for the whole processes. Other forwarding mechanisms like CATN [14], SCAN [15], and intra-AS [16] are either designed for edge networks only or utilize a global index exchange which incurs high overhead and limits the scalability. In addition, the forwarding mechanisms like SCAN are designed to work in parallel with IP routing; without IP-overlay routing, they cause unstable communication and large control overhead. Those schemes are not designed for native ICN forwarding and are not applicable for IoT.

CC-WSN [17] is a lightweight version of CCNx for resource-constrained devices in IoT by modifying the message format and naming scheme. NDN-WSN [7] is designed with two forwarding modes including flooding mode and directive mode with a flexible mode shift for IoT devices. Above forwarding mechanisms mainly focused on energy conservation techniques including broadcast storm avoidance, energy weight factors, and packet suppression to preserve energy consumption of IoT devices. In NETWRAP [18], the authors developed a wireless recharging scheme for NDN-based WSNs. In particular, the network is divided into several partitions, each partition with a cluster head. NETWRAP uses an energy Interest packet to collect residual energy information of nodes, which is then used for cluster head selection.

In [19], the authors reviewed NDN-based forwarding and routing mechanisms for wireless ad hoc networks. By considering critical challenges, reactive flooding is utilized to discover content providers instead of proactive routing. The issue is then considered in [20] using spatial network cascading failures. In [5], the authors conducted various experiments with NDN in IoT. However, the work lacks consideration of energy efficient design and flooding scope control [4], so it may under-perform for resource-constrained IoT devices. In [21], the authors designed HFHN, a hierarchical and flat based hybrid naming for building management, which is then used for packet forwarding. In [22], Ullah et al. proposed a push-based naming scheme for broadcast packet controlling in smart building and vehicular network use cases. The authors designed a hierarchical namespace for content as well as IoT devices to support minor modification of data packets. However, the design is not applicable for pull-based communication in IoT.

To address the above limitations, Muhammad et al. [23] proposed CIDF-WSN which uses a neighbor information base (NFIB) to enable IoT nodes to select the better next hop to forward an Interest packet. However, the Interest packet is still forwarded mainly based on FIB. This can lead to inefficient cases as discussed below. To address the limitations of existing works, this paper highlights the importance of CS information in an ICN forwarding mechanism. In addition to the use of FIB, our proposed scheme enables CS information in the data plane and uses CS information in the forwarding engine to optimize the forwarding efficiency for Interest packets. In comparison with existing approaches, the proposed scheme exploits CS to increase the cache hit ratio, thus improving the network performance instead of using only FIB.

## 3. The Proposed CS-Based Forwarding Scheme

### 3.1. Problem Statement

**Figure 1:**
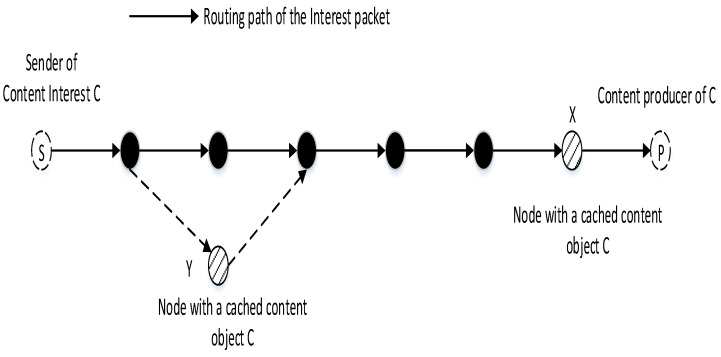
Problem illustration.

In existing ICN design [2,3], nodes forward Interest packets mainly based on forwarding information base (FIB). FIB is constructed with name prefixes registered by content producers and a list of next hops toward corresponding content producers. ICN forwarding engine uses that information to forward Interest packets towards corresponding content producers. In Figure 1, the Interest packet for content C from the sender S is forwarded toward the content producer P using FIB. Following existing ICN forwarding mechanisms, the Interest packet is forwarded along the path. At each intermediate node, the node (i.e., node X) processes search its CS for the requested content. If its CS contains the requested content C, the node returns the content to the sender. If there is no caching of C, the node processes check the pending Interest table (PIT) and then forward the Interest packet to the next hop using FIB.

The limitation of the existing design is that the cached content is exploited only if it is cached at a node on the forwarding path toward the corresponding content producer. If a sender requests the same content C in the former and the content is cached at a node (i.e., node Y) that is not on the path from S to P, the cached content C at Y does not benefit the request of S, although Y is close to S. This is inefficient packet forwarding, especially for resource-constraint IoT devices. In addition, as CS information is not shared among neighbor nodes, several neighbor nodes may cache the same content objects. As a result, this lowers the number unique content objects can be cached within a neighborhood of a node because the storage capacity of IoT device is limited. This paper proposes an efficient design to make CS information of a node available in the data plane to share with neighbor nodes. Based on that, we implement an efficient forwarding mechanism to improve the cache hit ratio, reduce the hit distance, network delay, and increase energy efficiency for IoT devices.

### 3.2. An Efficient Design for CS Information Transmission

As we discuss above, CS information of a node can help improve the efficiency of forwarding mechanism and caching policy of its neighbor nodes. However, the amount of CS information is normally quite large and inefficient for exchanging among IoT nodes. Transmitting the whole CS information can lead to another inefficient problem. Therefore, an efficient design for CS information exchange is required to take benefits of CS information in the data plane.

For that, we use the counting bloom filter (CBF) [24] in our design. A bloom filter is a space-efficient probabilistic data structure consisting of an m-bit array that is used to test whether an element is a member of a set. A CBF of a node is used to summarize a compact set of representing lists of content names cached in the CS of a node. We use CBF for storing and exchanging content name lists, instead of the full lists of content names because bloom filter (BF) is a space-efficient random data structure supporting the membership queries. In addition, we use CBF instead of the standard BF because CBF are represented by an array of m-bit counters supporting the deletion of elements as discussed in detail in [24]. This means that, compared to the bloom filter, to enable deletion of elements, the counting bloom filter uses an array of m counters instead of bits. This feature is required in IoT because content can be added and deleted from a CS.

Local CS information of a node is summarized using a CBF, namely a local CBF. Note that all CBFs have the same size. Local CBF of a node is updated when a content is added or deleted. Each node advertises its local CS information summary through a compressed local CBF [25] to the neighbors only to minimize the amount of information required to be transmitted. The idea behind compressed BF is that the algorithm takes advantage of choosing a parameter k to compress the m bit array and reduce the transmission size. The information is then piggybacked into the signaling advertisement message of the lower layer protocol as implemented in our previous study [26], so CS information transmission does not incur extra packet transmission, in order to save energy. In particular, as shown in our previous work, the information is inserted into signaling messages at the 802.15.4 MAC layer of the sender and decapsulated at the receiver side.

### 3.3. Neighbor Content Store Table

In addition to existing data structures of ICN [3,7] including Pending Interest Table (PIT), Forwarding Information Base (FIB), and Content Store (CS) of NDN’s forwarding engine, we design a new neighbor content store (N-CS) table for the proposed forwarding scheme. An N-CS table at a node is a compact table containing a number of counting bloom filters (CBFs) corresponding with the number of communication interfaces of the node. A CBF is used for an interface, as a compact set representing a list of content names cached in the CS at neighbor nodes corresponding with the interface, namely a neighbor CS. In our proposed forwarding scheme, each node advertises its CS information to neighbors using the efficient method described above. Other tables such as content store, pending interest table, and forwarding information base follow the existing design of the conventional ICN [2,6].

### 3.4. Forwarding Scheme

**Figure 2:**
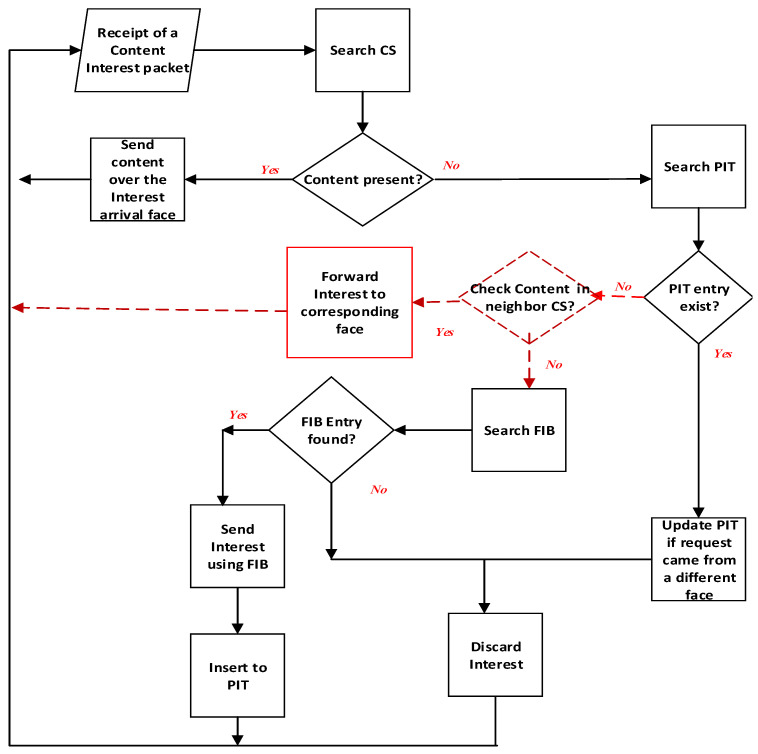
Forwarding engine of the proposed scheme compared to NDN.

The detailed procedures for Interest packet forwarding of the proposed scheme are presented in Figure 2. New procedures implemented in our proposed scheme are highlighted in red on the top of the conventional ICN forwarding engine [2,3] in black. When a node receives a content Interest packet, the node first checks its local CS. If the requested content is cached at the local CS, the node returns to the sender with its cached content object. If the node does not have the requested content, it checks its pending Interest table (PIT). The node then rejects the Interest packet if its PIT already contains the Interest or adds the Interest to its PIT if its PIT does not contain the Interest. These procedures follow the conventional ICN forwarding.

The node then checks its neighbor CS. If the requested content is cached at the neighborhood, the node forwards the Interest packet to the corresponding face toward the neighbor having the cached content object. The neighbor node receiving the Interest packet responds with its cached content object. Otherwise, the node forwards the Interest packet to the next hop using FIB toward the content producer. The above operations are processed recursively until the Interest is satisfied.

In this paper, we implement one hop CS based forwarding, but it can be extended for multiple hop neighbors (i.e., N hops, with N is the hop count). For multi-hop cases, we also use the neighbor CS table for each node and a CBF is used for an interface. However, a CBF is used to summarize a compact set of representing lists of content names cached by its N hop neighbors, instead of one hop neighbors. For that, the CS information summarized using CBF of a node is propagated to multiple hop neighbors instead of one hop neighbors. Other operations remain the same.

For Interest packet forwarding, after the phase in which a node A checks PIT for Interest packet forwarding, the node then checks if the packet forwarding distance using FIB is smaller than N (i.e., hop count), and the node forwards the Interest packet using FIB for efficiency. Otherwise, in the phase in which a node checks its neighbor CS table for Interest packet forwarding, if the requested content name exists in a CBF of an interface in the neighbor CS table, the node adds a hop counter to the Interest packet and forwards the Interest to next hops through the corresponding interface until it is satisfied by CS of a neighbor. At each hop, the hop counter is increased by one. When the Interest packet is received by a node containing the cached content object, the node returns to the sender with its cached content object. In cases of errors when the requested content is not found within N hops, a fall-back recovery is performed by sending a notification ACK back to node A. FIB-based NFD forwarding is then executed at node A. The above operations are processed recursively until the Interest is satisfied.

## 4. Performance Evaluation

### 4.1. Implementation and Configuration

We implement the proposed CS-based forwarding scheme in Contiki [6]. We then perform simulations using COOJA simulator [6] with 1050 nodes in a coverage area of 1000 × 1000 m. Nodes are randomly deployed with the sensing correlation of the nodes obtained from the IntelLab sensor data [27]. Among these nodes, 100 nodes are IoT content producers, 200 nodes are cache nodes, and 750 routing nodes. The system generates 3000 content requests from random nodes following Zipf-like distribution with the value of α between 0.2 and 1. The CS storage capacity of each cache node is varied from from 5 to 25 content objects.

Due to resource constraints, we conduct experiments using nodes with light-weight sensing information and limited storage capacity. As implemented in our prior study [26], we reuse HTTP-CoAP converter in this paper to convert application requests in HTTP to CoAP for IoT nodes. Application requests are encoded using templates in extensible markup language (XML) and decoded using SensorML interpreter for IoT nodes [28]. For data collection, we utilize CTP and LPL [26] as the data collection schemes and 802.15.4 MAC (Media Access Control) mechanism. We use closest-fit-pattern matching (CPM) as the radio noise model [26]. We use a CCA (clear channel assessment) [26] check parameter up to 400 times. The detailed parameter configurations of simulations are shown in Table 1. Other parameters remain the same as the default configurations of the Contiki CC2420 radio model [26]. By default, we use the cache size of 20 content objects, wakeup interval of 1 s, and one hop neighbor CS information if those parameters are not specified. The naming scheme [29] is used for IoT nodes. Key modifications of the proposed scheme implementation in comparison with the existing CCN/NDN implementation are presented Section 3.4. The experimental results are reported at a 96% confidence interval. We present the performance evaluation of the proposed scheme in comparison with state-of-the-art Interest packet forwarding schemes in IoT, CIDF [23] and the conventional ICN forwarding [2,3].

### 4.2. Obtained Experimental Results

We use the following metrics for the performance evaluation and comparison.

**Average radio duty cycle**: We use average radio duty cycle as an indicator for energy efficiency [30]. We consider timing aspects for calculating the duty cycle (e.g., time for transmission). A radio duty cycle of a node is the ratio of radio active period and the cycle time, the cycle time is the duration of active time and the sleep time of IoT nodes. The overall duty cycle (DC) of a node *i* is calculated using (1) by simply adding duty cycles for each radio operation: listening ($DC_{lx}$), transmitting ($DC_{tx}$), receiving ($DC_{rx}$), overhearing ($DC_{over}$), and additional operations ($DC_{add}$) [30]:

(1) $$DC_{i}=DC_{i}^{lx}+DC_{i}^{tx}+DC_{i}^{rx}+DC_{i}^{over}+DC_{i}^{add}$$

To measure the radio duty cycle, we record changes in the radio’s states and use a counter to accumulate the time period used in each state. At the end of simulation, we calculate the average radio duty cycle and report average results.

Average duty cycle of nodes in a network is calculated as follows:

(2) $$DC_{average}=\frac{\sum_{i=1}^{n}DC_{i}}{n}$$

where *n* is the total number of IoT nodes.

**Average cache hit ratio**: measures the percentage of content interest that are satisfied by cached content objects (COs) in the CS of IoT nodes. This metric implies that the higher the cache hit ratio, the higher the energy efficiency of IoT nodes that is achieved. As a result, this metric also reflects the network performance of the forwarding mechanism. We calculate the average cache hit ratio as follows:

(3) $$CH_{average}=\frac{\sum_{i=1}^{n}c_{i}}{p}$$

where $c_{i}$ is the total number of content objects provided by CS of node *i* and $p=\sum_{i=1}^{n}p_{i}$ is the total number of content objects provided by nodes responding to content interests.

**Average content retrieval latency**: indicates the average time needed for interest packet get satisfied whether from an original content publisher or from a cache. A good forwarding decision should reduce the content retrieval latency. The average content retrieval latency is calculated as follows:

(4) $$L_{average}=\frac{\sum_{c=1}^{p}L_{c}}{p}$$

where $L_{c}$ is the latency to retrieve content c, and *p* is the total number of content objects provided by nodes responding to content interests.

**Response ratio R**: measures the ratio between the total number of content objects responded *p* and the total number of Interest packet sent by nodes *I* in the network. This metric shows the reliability of the network. The response ratio is calculated as follows:

(5) $$R=\frac{p}{I}=\frac{\sum_{i=1}^{n}p_{i}}{\sum_{i=1}^{n}I_{i}}$$

#### 4.2.1. Energy Efficiency

**Figure 3:**
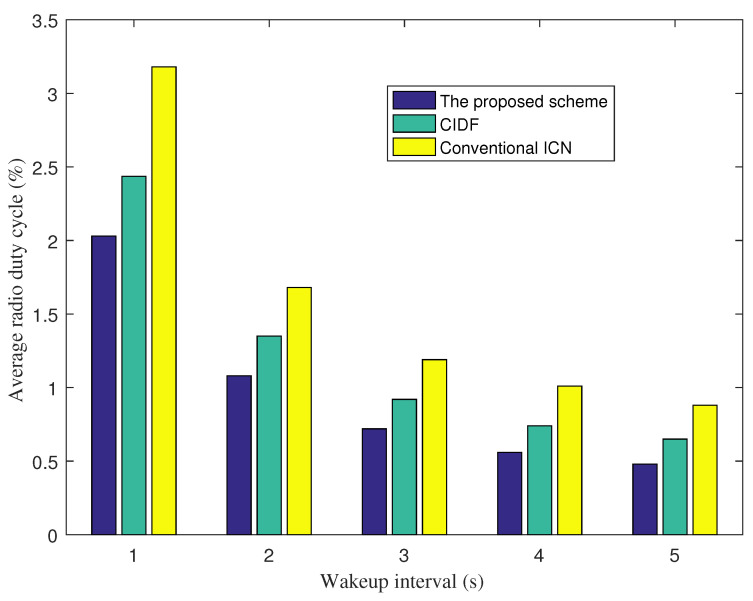
Average radio duty cycle vs. wakeup intervals.

Figure 3 shows the average radio duty cycle of nodes in the network under various wakeup interval values. In all wakeup interval cases, the proposed scheme achieves a lower average duty cycle than CIDF and the conventional ICN. This implies that the proposed scheme improves the energy efficiency of IoT nodes significantly compared to CIDF and the conventional ICN. This is a result of the efficient Interest packet forwarding and content object forwarding of the proposed scheme by exploring content store information of nodes to find better routes. An interesting phenomenon we obtain is that the higher the wakeup interval that is set, the better the improvement ratio of energy efficiency that is achieved. In particular, the energy efficient improvement ratios of the proposed scheme compared to CIDF are 25.2%, 28%, 31.5%, 35.2%, and 42.8% corresponding with the wakeup interval of 1 s, 2 s, 3 s, 4 s, and 5 s, respectively. When the wakeup interval increases, nodes wake up less regularly. Therefore, it can be more costly to forward an Interest in multiple hops. By exploring CS information of nodes to find cached content objects, the proposed scheme helps reduce the forwarding distance for Interest packets. As a result, Interest packets can be satisfied by cached content objects at neighborhood, instead of forwarding them to content producers.

#### 4.2.2. Cache Hit Ratio

**Figure 4:**
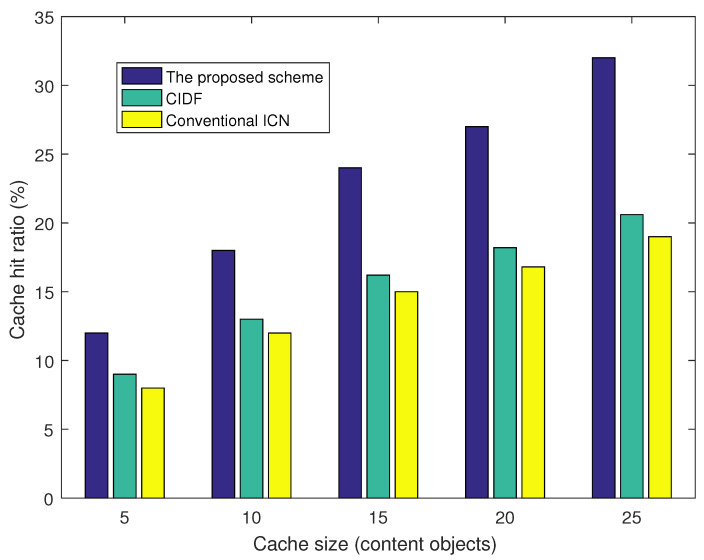
Average cache hit ratio vs. cache size.

Figure 4 presents the cache hit ratio of the three schemes under various cache sizes. The proposed scheme shows a higher cache hit ratio than CIDF and the conventional ICN because the proposed scheme actively utilizes neighbor CS information for Interest packet forwarding. We find that the greater the cache size that is used, the better the improvement ratio of cache hit ratio that is achieved. The proposed scheme achieves 33%, 40.2%, 48%, 51.8%, and 55.2% cache hit ratio improvement corresponding with the cache size of 5, 10, 15, 20, and 22 content objects, compared to CIDF. The reason is that, with a greater cache size, nodes can cache a higher number of content objects which are then explored by the proposed scheme for Interest packet forwarding. This feature is practical because the storage hardware becomes cheaper and cheaper by the time.

#### 4.2.3. Average Content Retrieval Latency

**Figure 5:**
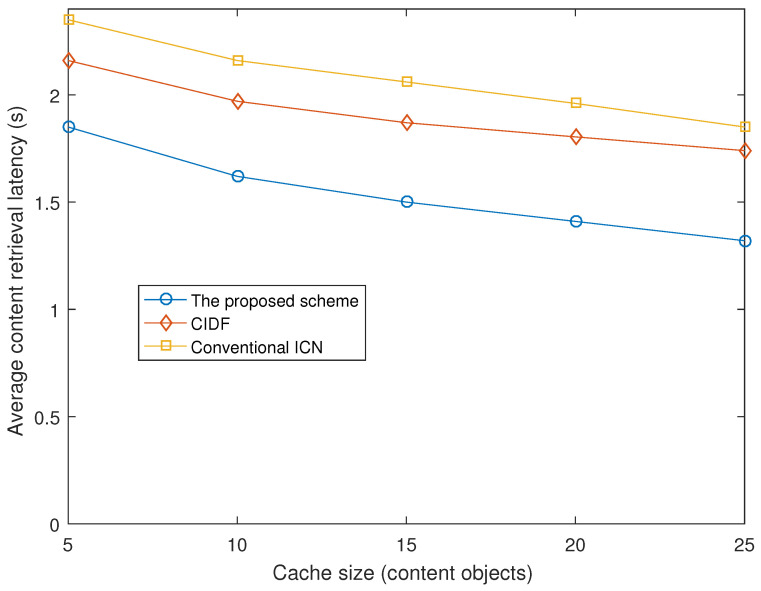
Average content retrieval latency vs. cache size.

The previous figure also indicates that the proposed scheme should have another benefit of average content retrieval latency because it improves the cache hit ratio significantly. Figure 5 depicts average content retrieval latency of Interest packets in the three forwarding schemes under various cache sizes. The proposed scheme improves the average content retrieval latency significantly compared to CIDF and the conventional ICN. We find that the greater the cache size that is used, the higher the latency improvement that is achieved with the proposed scheme.

#### 4.2.4. Response Rate

**Figure 6:**
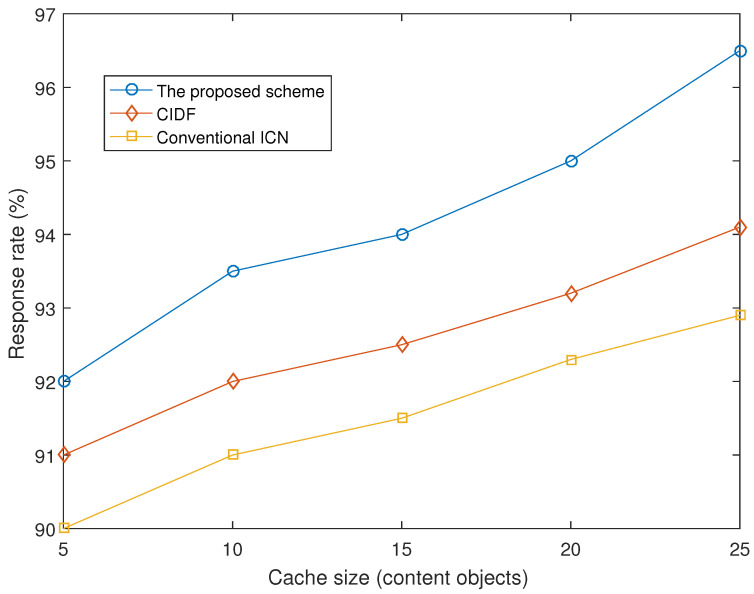
Response rate vs. cache size.

We also measure the response rate of Interest packets to understand the quality of service under different schemes. Figure 6 shows the response rate of the three forwarding schemes under various cache sizes. Although the response rate of all schemes are high, at above 90%, the proposed scheme achieves a better response rate when the cache size is increased from 5 to 25. In constrained IoT environments, failed request-responses can be due to many reasons such as interference, energy issues, and intermittent connection due to sleep-wakeup schedules. Therefore, reducing the forwarding distance of Interest packets also leads to increasing the quality of services.

## 5. Discussion and Conclusions

This paper proposes to enable neighbor CS information in the data plane of ICN to improve the Interest packet forwarding, especially in IoT environments. For that purpose, we carefully design an efficient way for CS information sharing using CBF. We extend the existing ICN forwarding based on FIB with neighbor CS information to optimize the forwarding efficiency for Interest packets. In comparison with existing approaches, the proposed scheme exploits CS to increase the cache hit ratio, thus improving the network performance instead of using only FIB. We implement the proposed scheme and compare with state-of-the-art forwarding schemes in IoT. Obtained results show that the proposed scheme achieves a significant improvement in terms of energy efficiency, cache hit ratio, content retrieval latency, and response rate. In future works, we plan to collaborate various caching mechanisms with the forwarding scheme to enhance the network performance and perform experiments with edge cloud computing to integrate the forwarding scheme with our existing systems. 

## Figures and Tables

**Table 1:** Parameters.

Parameter	Value	Parameter	Value
Number of nodes	1050	CCA check	400 times
α	0.2–1	cache size p	5–15 objects
Wakeup interval	1–5 s	MAC	LPL
radio	CC2420	noise model	CPM

## Data Availability

Not applicable.

## Abbreviations

The following abbreviations are used in this manuscript:

**Acronym**	**Meaning**
CS	content store
CDN	content delivery networks
PIT	pending interest table
BF	Bloom filter
CBF	counting Bloom filter
N-CS	neighbor content store
CPM	closest-fit pattern
NDN	named data networking
CCN	content centric networking
CO	content object
